# To the Medical Profession of Texas

**Published:** 1898-09

**Authors:** 


### Society Notes.

### To The Medical Profession of Texas.

BY THE COMMITTEE ON MEDICAL LEGISLATION.

The modern facilities for teaching medicine and the opportunities now within reach of every student make it not only unnecessary but a crime to turn loose ignorant and incompetent medical men upon an unwary and too susceptible public. American medicine should not be disgraced any longer by illiterate physicians. The enlightened people of every State should, in self protection, demand that their health and lives be placed only in the hands of well trained and educated medical men.

The people themselves can have no knowledge nor suspicion of how many eyes, ears, limbs or even lives are sacrificed upon the altar of ignorance and superstition as a result of insufficient laws, nor do they know of the great advance constantly being made in medicine, and the necessity for physicians being well informed upon the subject of all new discoveries of value. Every country where civilization exists has, or is making, provision for giving their people better qualified doctors. Nearly every State and territory in the union has a law upon its statute books governing the practice of medicine. It is a stigma upon the fair name of Texas that she should be of the last to make so wise a provision for the protection of the health and lives of her citizens; there is no reason for it. The major part of the opposition comes from quacks and charlatans who have been driven from other States and who are flocking into this. These laws are not in any degree a hindrance or a hardship to any except the ignorant who should have no place in the ranks of such a profession, nor be trusted with the lives of human beings.

The formation of the National Confederation of the State Medical Examining Boards which has existed for several years is evidence of the progress being made in this direction. Texas can have no place in this Confederation until she has enacted a law placing her on an equality with all other States that have State boards of examiners. Conscientious teachers all admit that the State examinations have proved a stimulus to both students and teachers, and since elevating the standard of medical education, the more respectable and best equipped schools have a more intelligent and better educated class of students. All students who are ambitious and desire to be respectable in medicine want the best. It requires more time and a little more expense but serves them more satisfactorily in their early struggles for recognition and professional favor, as well as maintaining a high position in after years.

The States are helping the schools to elevate and maintain their requirements for matriculation and graduation by compelling every person, irrespective of school or dogma, to prove his qualifications by passing an examination upon the fundamental principles of medicine. No partiality is shown to anyone, it matters not what may be the cry of quacks and pretenders; they have equal rights with all others if they possess the requisite knowledge. There should be in every State a definite standard equally with all other States for admission to the study of medicine, equalization in the preliminary requirements of medical students and a uniform rule of applying the tests. If a person's mind has not already been disciplined to the extent of acquiring a good English education before he begins the study of medicine, it presents a slender foundation upon which to engraft a knowledge of such a multiform science and art. As civilization develops, immunity from disease lessens, hence vigilance and efficiency must be greater, the period of preparation longer, and training increased in length, depth and breadth. The foundation principle for a separate examination for license to practice medicine is that it shall apply to all with absolute impartiality; there must be no exceptions to the rule and no exemptions. But a law on this subject cannot be made retroactive, and all legal practitioners in active practice at the time of its passage must continue to be so recognized. If legislators could be made to appreciate the fact that public health interests are involved in the question of State license, that every attempt to weaken the principal is a blow at public sanitation, and that higher standards of medical education mean better health for the people, surely not one would hesitate to vote for such a law.

The Texas State Medical Association has unanimously adopted a bill governing the practice of medicine, to be recommended to our next legislature for enactment, that is just and fair to all, and no reasonable person can take exceptions to it. It is a constitutional bill, and gives every person who has received a good, modern medical education and passes a satisfactory examination the right to practice medicine, surgery or midwifery in Texas. It cannot interfere with those who are now legal practitioners. It does not question, restrict or in any way interfere with any person's belief or dogma. It only requires that every person who applies for license shall have a good medical education and that he passes a satisfactory examination before the board of his own selection. The same questions are required for all in those branches absolutely necessary for the foundation of a practical medical education, about which there is no dispute and which all sects and schools profess to teach; in this matter it is entirely fair to all, no partiality or advantage can be shown. In materia medica, practice and therapeutics the applicant selects that board whose tenets agree with his own and is examined by it alone. This bill establishes a medical council to be composed of State officers, viz.: the attorney-general, the Texas State health officer, the senior medical member of the board of regents of the University of Texas, and the presidents of the State boards of medical examiners provided for in the bill.

Its duties are plain and cannot be partial to any applicant for license. It is composed of men representing the legal profession and the medical profession—regular—the homeopathic and the eclectic. All applications for examination are made to the council, which issues a permit to the applicant to appear before the board of his own selection for examination. This council selects the questions for examinations from lists submitted to it by the three boards, which in anatomy, physiology, histology, pathology, surgery, obstetrics, gynecology, hygiene and medical jurisprudence shall be the same for all applicants, the same standard of qualifications in these branches being required for all. This prevents any unfairness or partiality, and no complaint can be made by any applicant, for all receive the same treatment. No applicant need be known personally by the board or council, as examination papers are signed by number or motto and not by the name of the person examined.

The council examines the ratings and decisions of the respective boards and sees that they are fair and just. It issues a license to each successful applicant; said license to have affixed to it the seal of the State of Texas, the signatures of the members of the council and of that board which conducted the examination.

The bill provides for three boards of medical examiners consisting of seven members each, viz.: The board of medical examiners of the [Medical Association of the] State of Texas, the homeopathic board of medical examiners of the State of Texas, and the eclectic board of medical examiners of the State of Texas. These boards will be appointed by the governor from a list of names twice the number necessary for appointment, to be recommended by the Texas State Medical Association, the Homeopathic Medical Association of Texas, and the Eclectic Medical Association of Texas. The reasons for the recommendations of the men for such positions is obvious; the governor is not in position to know who is competent to fill the important position of examiner on either board. These various societies know better than any one else and it is proper that they should select a list of such persons as they deem most competent and suitable. The governor is not limited to the number that compose the boards, but has twice the number to select from. The boards will be required to qualify by taking the oath of office as all other State officers. The bill provides for the same number for each, the same duties, the same privileges and the same requirements for all, therefore there can be no reasonable or just complaint or opposition to its provisions. This bill requires that all applicants for license shall present satisfactory evidence of having a good English education and a good medical education from a chartered medical school of good reputation and standing, having an adequate standard of preliminary requirements and power to confer degrees.

It is not generally known that a great number of lives of women and children are lost and a great many are made invalids for life by the ignorance and incompetency of midwives; they infect many women by the lack of proper preparation of their hands and dressings with no regard for the antiseptic requirements of the present day. Obstetrics is a science that has progressed with all other departments of medicine and its successful practice requires careful study and thorough training in order that many sadresults which now obtain may be prevented. It is, therefore, of supreme importance that only well trained midwives should be permitted to practice the art. This bill provides for the examination of all who propose to practice midwifery, and after passing a satisfactory examination they are licensed to practice the same. As the enactment of this law will require no appropriation from the State, would be a decided benefit to the people and at the same time, to all honest medical practitioners, it is difficult to see why it should be opposed by any except ignorant pretenders and quacks. A proper presentation of this subject in a fair and honest way to all practitioners and to all candidates for the Legislature will undoubtedly meet with favor.

A thorough and earnest canvas of the candidates throughout the State in its behalf cannot fail to elicit for it commendation and enactment into a law next winter.

We urge upon every member of our beloved profession who has the good of mankind at heart to make of this a personal matter. Let each and every physician see the candidate for the State senate and lower house in his district, explain to them the scope and purposes of the bill and we feel assured that the result of this duty well done, as it deserves to be, will be the passage of this act at the next session of the legislature.

M. M. Smith,
S. E. Hudson,
J. T. Wilson,
Committee.

